# Effects of short-term treatment with atorvastatin in smokers with asthma - a randomized controlled trial

**DOI:** 10.1186/1471-2466-11-16

**Published:** 2011-04-07

**Authors:** Georgina Braganza, Rekha Chaudhuri, Charles McSharry, Christopher J Weir, Iona Donnelly, Lisa Jolly, Jane Lafferty, Suzanne M Lloyd, Mark Spears, Frances Mair, Neil C Thomson

**Affiliations:** 1 Respiratory Medicine, Institute of Infection, Immunity & Inflammation, University of Glasgow, Glasgow, UK; 2 Immunology, Institute of Infection, Immunity & Inflammation, University of Glasgow, Glasgow, UK; 3 MRC Hub for Trials Methodology Research, University of Edinburgh, UK; 4 Robertson Centre for Biostatistics, University of Glasgow, Glasgow, UK; 5 General Practice, University of Glasgow, Glasgow, UK

## Abstract

**Background:**

The immune modulating properties of statins may benefit smokers with asthma. We tested the hypothesis that short-term treatment with atorvastatin improves lung function or indices of asthma control in smokers with asthma.

**Methods:**

Seventy one smokers with mild to moderate asthma were recruited to a randomized double-blind parallel group trial comparing treatment with atorvastatin (40 mg per day) versus placebo for 4 weeks. After 4 weeks treatment inhaled beclometasone (400 μg per day) was added to both treatment arms for a further 4 weeks. The primary outcome was morning peak expiratory flow after 4 weeks treatment. Secondary outcome measures included indices of asthma control and airway inflammation.

**Results:**

At 4 weeks, there was no improvement in the atorvastatin group compared to the placebo group in morning peak expiratory flow [-10.67 L/min, 95% CI -38.70 to 17.37, p = 0.449], but there was an improvement with atorvastatin in asthma quality of life score [0.52, 95% CI 0.17 to 0.87 p = 0.005]. There was no significant improvement with atorvastatin and inhaled beclometasone compared to inhaled beclometasone alone in outcome measures at 8 weeks.

**Conclusions:**

Short-term treatment with atorvastatin does not alter lung function but may improve asthma quality of life in smokers with mild to moderate asthma.

**Trial Registration:**

**Clinicaltrials.gov identifier: **NCT00463827

## Background

Smokers with asthma have poor symptom control, accelerated decline in lung function and an attenuated response to inhaled corticosteroids compared to non-smokers with asthma[1-4]. Optimum medical therapy for smokers with asthma has not been established. There is an unmet need for alternative or additional drugs for smokers with asthma who are unable to stop smoking.

In addition to reducing cholesterol biosynthesis by inhibiting 3-hydroxy-3-methylgluteryl coenzyme A (HMG-CoA) reductase, statins have pleiotropic anti-inflammatory effects[5] that may be beneficial in the treatment of chronic inflammatory diseases[5,6]. Preclinical *in vitro *and *in vivo *studies, including experimental models of allergic lung inflammation[7,8], have shown that statins reduce components of airway inflammation potentially relevant to the pathogenesis of asthma. Despite preliminary data showing an absence of clinical benefit with short-term statin therapy on asthma symptoms or lung function in non-smokers with asthma[9-11], there is indirect evidence to suggest that statins may show efficacy in patients with asthma who smoke. First, simvastatin inhibits lung parenchymal destruction, peribronchial and perivascular inflammatory cell infiltrate and leads to a reduction in levels of matrix metalloproteinase-9 in a rat model of smoking related lung disease[12] and reduces airway inflammation and reverses elastase-induced pulmonary emphysema in mice[13]. Second, statins enhance corticosteroid sensitivity in asthma[14,15], possibly by restoring histone deacetylase (HDAC) activity[16], which is reduced by cigarette smoking[17]. Thirdly, retrospective or case cohort studies have shown statin use is associated with a reduced decline in lung function in cigarette smokers[18] and reduced exacerbation rates, hospital admission and mortality in patients with COPD[19]. Taken together, this evidence suggests that statin treatment may improve asthma control in smokers with asthma. This study aimed to test the hypothesis that short term treatment with atorvastatin, either alone or in addition to low dose inhaled corticosteroid improves lung function or other indices of asthma control and/or airway inflammation in smokers with asthma. Atorvastatin was chosen because of its favourable *in vitro *and *in vivo *anti-inflammatory properties [20-22] and evidence of clinical benefit at the dose of 40 mg daily in rheumatoid arthritis [21].

## Methods

### Study subjects

Cigarette smokers with chronic asthma symptoms of more than one year duration, aged 18 to 60 years were eligible to take part. Participants were regular smokers with a history of 5 pack years or more. Patients were recruited from general practice, hospital clinics and research databases. Smoking cessation advice was offered at the study screening visit and eligible subjects who were not willing to quit were enrolled. Patients were excluded if they were already taking a statin or medications known to interact with statins such as antifungal agents, macrolide antibiotics, cyclosporin, gemfibrozil, verapamil and amiodarone. All subjects demonstrated reversibility in FEV_1 _following salbutamol of ≥12% and ≥200 ml or PC_20 _≤ 8 mg/ml or ≥ 20% variability in peak expiratory flow (PEF)[23]. The West Glasgow Research Ethics Committee approved the study and all patients gave written informed consent.

### Study design

The aim of the study was to test the hypothesis that short-term treatment with atorvastatin improves lung function or indices of asthma control or airway inflammation in smokers with asthma. Patients had been free of exacerbation and lower respiratory tract infection for a minimum period of 6 weeks prior to randomization. Where relevant, inhaled corticosteroid (ICS) and long-acting bronchodilator (LABA) treatment were weaned over a 4-10 week period. On completion of a two-week run in period free from ICS and LABA, a baseline visit was performed. Subjects recorded their PEF and asthma symptoms twice daily throughout the study. Randomization was conducted via an interactive telephone service (IVRS) in randomized blocks of length four. Patients were assigned to eight weeks of atorvastatin 40 mg daily or a matched placebo. After four weeks inhaled beclometasone (Clenil^® ^Modulite^® ^200 μg twice daily) was also commenced in both randomized groups. Treatment with short-acting β-_2 _agonist was allowed throughout the study. At baseline, 4 and 8 week visits, electronic PEF data were downloaded and spirometry performed. At these visits patients completed a validated asthma control questionnaire (ACQ) and asthma quality of life questionnaire (AQLQ); induced sputum was performed and exhaled nitric oxide was measured. At baseline and 8 weeks, blood samples were taken for immunological tests, lipids and liver transaminases.

### Measurements

PEF was recorded using Piko-1 electronic peak flow meters (nSpire Hertford UK) and symptoms were recorded in a validated diary card[24]. ACQ[25] and AQLQ [total and individual domains][26] results were also recorded. The total AQLQ score is the mean of all 32 responses and the individual domain scores are the means of the items in those domains. A change in score of 0.5 is the smallest change that is considered clinically important. Exhaled nitric oxide (NO) levels were measured at 50 ml/s in concordance with standardised guidelines[27] (Niox Flex, Aerocrine, Sweden). Spirometry was performed to American Thoracic Society guidelines. Exhaled carbon monoxide levels were measured by hand held Smokerlyser. (Bedford scientific, UK). Airway hyper-responsiveness was measured by Cockcroft's methacholine challenge test with concentrations from 0.03 mg/ml to 16 mg/ml[28]. Blood samples were processed by local accredited laboratories for urea and electrolytes, liver transaminases, thyroid function, creatinine kinase levels and lipid profile; haematological testing (full blood count), and total IgE and specific IgE to cat, house dust mite and grass. Total IgE levels >120 IU/ml was considered elevated and specific IgE levels >0.35 AU/ml was used to define atopic status. Sputum induction was performed as previously described[29], using a low concentration of DTT (0.003%) to disperse cells without undue effects on mediator measurements. Sputum supernatant fluid was analysed for leukotriene (LT) B_4 _and myeloperoxidase (MPO) using enzyme immunoassay (EIA) (LTB_4 _from R&D Systems, Abingdon, UK, MPO from Cambridge Bioscience, Cambridge, UK.), and interleukin (IL)-2, 4, 6, 8, 10 and tumor necrosis factor (TNF)-α using a Luminex microbead fluorescence kit (Biosource, Invitrogen, Paisley, UK). Serum was analysed for high sensitivity (hs)-CRP by EIA (R & D Systems) and sICAM-1, E-selectin, P-selectin, IL-2, 4, 6, 8, 10 and TNF-α using a multiplex fluorescence bead kit (sICAM-1, E-selectin and P-selectin from Merck Chemicals, Hertfordshire, UK. Interleukins and TNF-α by Biosource). Current smoking was confirmed by serum cotinine measurement (Cozart, Oxfordshire, UK). Treatment compliance was assessed by tablet count and inhaler weight. Adverse event information was reviewed at each study visit.

### Analysis

Baseline characteristics were described by number and percentage of patients for categorical variables and mean (SD) or median and inter-quartile range for normally distributed or skewed continuous variables respectively. The full analysis set was defined as all randomized subjects with at least one post-baseline assessment of PEF (primary endpoint). The primary endpoint was calculated as the mean of the morning PEF measurements from the Piko-1 electronic peak flow meter recordings during the seven days immediately preceding each study visit. If fewer than three days of data were recorded within that period, patient diary entries for morning PEF were substituted in place of the missing values. The mean was then calculated if there were at least three days of data available, otherwise the response was taken to be missing. Response to atorvastatin on lung function, diary data, induced sputum, mediator levels and exhaled nitric oxide versus placebo was assessed by analysis of covariance, where the response was change from baseline and the baseline value was the covariate. All data were analysed using SAS version 9 (SAS Institute, Cary, NC). A sample size of 68 was calculated to have 80% power to detect a mean difference of 25 L/min in change from baseline to four weeks in morning PEF, the primary endpoint[30], assuming a standard deviation of changes of 36 L/min, using a two sample t-test with a 5% two-sided significance level. Recruitment of 74 patients was planned to ensure that 68 patients completed the study.

## Results

### Recruitment and baseline characteristics

**Figure 1:**
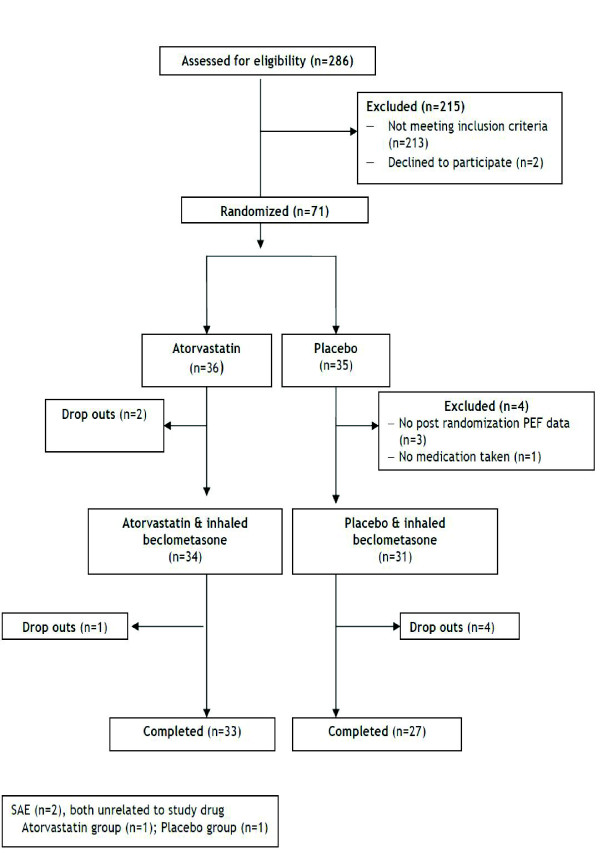
**Flow of patients through study**.

Of the 286 subjects contacted, 131 volunteers were consented and 71 were randomized to either the atorvastatin treatment group (n = 36) or the placebo group (n = 35) (Figure 1). Baseline demographic and clinical characteristics of patients are listed in Tables 1 and 2. The two groups were well balanced with respect to these characteristics.

**Table 1:** Demographics and clinical baseline characteristics

	Atorvastatin (n = 36)^†^	Placebo (n = 32)^†^
Age, years	40.4 (34.8,45.5)	43.0 (38.1,52.0)
Male sex, n (%)	15 (41.7%)	15 (46.9%)
Smoking history (pack years)	24 (14,34)	21 (10,30)
Duration of asthma symptoms, years	24.0 (14.5,32.5)	19.5 (12.5, 38.5)
Atopic, n (%)	18 (54.5%)	21 (67.7%)
Total IgE (IU/ml)	82 (19,192)	197 (76,517)
Use of inhaled corticosteroid at screening, n (%)	19 (52.8%)	16 (50.0%)
Equivalent beclometasone dose at screening, μg	100 (0,400)	200 (0,800)
Use of LABA at screening, n (%)	8 (22.2%)	14 (43.8%)
Pre-bronchodilator FEV_1 _% predicted	84 (71,98)	79 (65,92)
FEV_1 _% reversibility	11 (6,16)	12 (8,19)
Serum cotinine (ng/mL)	85 (78,90)	82 (79,94)

Definition of abbreviations: FEV_1_, Forced Expiratory Volume in one second; LABA, long-acting beta_2_-agonist

Data represented as median (IQR) unless specified.

**^† ^**Number of randomized subjects with at least one post-baseline assessment of PEF

**Table 2:** Clinical outcomes after atorvastatin treatment or placebo

Variables	Baseline		4 weeks		†Treatment difference, atorvastatin minus placebo (95% CI)	8 weeks		†Treatment difference, atorvastatin minus placebo (95% CI)
				
	Atorvastatin (n = 36)	Placebo (n = 32)	Atorvastatin (n = 34)	Placebo (n = 30)		Atorvastatin & ICS (n = 33)	Placebo & ICS (n = 27)	
**Morning PEF L/min**	381.33 (138.79)	348.99 (116.49)	376.39 (141.63)	357.65 (121.38)	-10.67 (-38.70, 17.37) p = 0.449	390.51 (123.38)	354.64 (123.70)	10.68 (-20.29, 41.65) p = 0.492

**Evening PEF L/min**	391.9 (137.1)	375.8 (118.4)	384.8 (137.1)	367.4 (117.2)	4.95 (-18.61, 28.51) p = 0.676	400.0 (140.1)	368.3 (130.5)	25.03 (-2.04, 52.11) p = 0.069

**FEV_1 _pre-salbutamol, L**	2.76 (0.91)	2.51 (0.76)	2.79 (0.83)	2.59 (0.74)	-0.01 (-0.13, 0.11) p = 0.872	2.80 (0.89)	2.63 (0.76)	0.03 (-0.10, 0.16) p = 0.651

**FEV_1 _post-salbutamol, L**	3.08 (0.94)	2.88 (0.75)	3.02 (0.88)	2.89 (0.75)	-0.03 (-0.13, 0.07) p = 0.492	3.02 (0.90)	2.93 (0.74)	-0.02 (-0.12, 0.07) p = 0.642

**PC_20 _methacholine** **geometric mean, mg/ml**	3.29 (4.03)	2.15 (3.34)	4.12 (4.86)	2.93 (5.0)	1.36 (-1.29, 4.01) p = 0.307	5.31 (6.2)	2.31 (4.0)	1.37 (-0.65, 3.40) p = 0.179

**ACQ Score** **[6 questions]**	1.86 (1.02)	1.86 (0.82)	1.42 (0.68)	1.91 (1.10)	-0.48 (-0.84,-0.12) **p = 0.009**	1.29 (0.93)	1.64 (1.10)	-0.37 (-0.8,0.07) p = 0.096

**ACQ score** **[7 questions]**	1.88 (1.00)	1.96 (0.79)	1.50 (0.67)	1.90 (0.90)	-0.37 (-0.67, -0.07) **p = 0.016**	1.38 (0.93)	1.77 (1.00)	-0.35 (-0.74, 0.04) p = 0.081

**AQLQ** **Total score**	5.36 (1.05)	5.14 (1.14)	5.71 (0.83)	5.04 (1.30)	0.52 (0.17, 0.87) **p = 0.005**	5.79 (1.02)	5.27 (1.34)	0.37 (-0.02, 0.76) p = 0.063

**AQLQ Symptoms Domain**	4.96 (1.08)	4.82 (1.20)	5.46 (0.90)	4.74 (1.39)	0.63 (0.18, 1.09) **p = 0.007**	5.67 (4.96,6.34)	4.88 (3.91,6.5)	0.42 (-0.05, 0.89) p = 0.082

**AQLQ Activity Limitations Domain**	5.84 (1.06)	5.52 (1.15)	5.99 (0.90)	5.36 (1.25)	0.40 (0.10, 0.71) **p = 0.010**	6.08 (0.97)	5.58 (1.29)	0.31 (-0.01, 0.63) p = 0.061

**AQLQ Emotional Functions Domain**	5.51 (1.36)	5.10 (1.35)	5.90 (1.19)	5.02 (1.62)	0.60 (0.10, 1.09) **p = 0.019**	5.87 (1.39)	5.30 (1.62)	0.24 (-0.26, 0.74) p = 0.335

**AQLQ Environmental Stimuli Domain**	5.10 (1.30)	5.09 (1.33)	5.41 (1.18)	5.07 (1.44)	0.34 (-0.09, 0.77) p = 0.123	5.71 (1.15)	5.13 (1.47)	0.55 (0.07, 1.03) **p = 0.026**

**Reliever inhaler puffs per 24 hrs**	3.19 (2.55)	2.94 (2.93)	2.58 (2.67)	3.97 (5.45)	-0.66 (-1.69, 0.37) p = 0.205	2. 13 (2.63)	1.99 (2.33)	-0.12 (-1.35, 1.11) p = 0.841

Abbreviations: ACQ, Asthma Control Questionnaire; AQLQ, Asthma Quality of Life Questionnaire; PEF, peak expiratory flow rate; FEV_1_, forced expiratory volume in one second; Data represented as mean (SD) unless otherwise indicated.

^†^Treatment difference from analysis of covariance, analysing change from baseline and adjusting for baseline value.

### Changes in clinical outcomes

**Figure 2:**
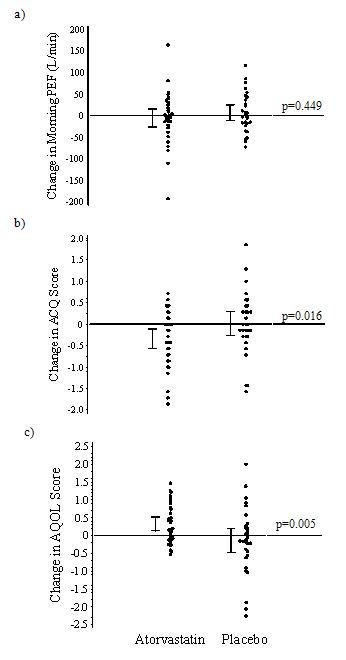
**Change from baseline for both treatment groups in (a) morning PEF [L/min], (b) ACQ score, (c) AQLQ total score at 4 weeks**. P values are for comparisons of the mean change from baseline between the two treatments.

Changes in clinical outcomes after atorvastatin treatment are listed in Table 2 and illustrated in Figure 2. At 4 weeks, the change in mean morning PEF, as compared with baseline, did not differ substantially between the atorvastatin and placebo treatment periods [mean difference -10.67 L/min, 95% CI -38.70 to 17.37, p = 0.449]. There was an improvement in the atorvastatin group compared to the placebo group at 4 weeks in the ACQ score [-0.37, 95% CI -0.67 to -0.07, p = 0.016] and AQLQ total score [0.52, 95% CI 0.17 to 0.87 p = 0.005] as well as AQLQ symptoms domain [0.63, 95% CI 0.18 to 1.09 P = 0.007], activity limitations domain [0.40, 95% CI 0.10 to 0.71 P = 0.010] and emotional functions domain [0.60, 95% CI 0.10 to 1.09) P = 0.019]. At 8 weeks there was no significant improvement with atorvastatin and inhaled beclometasone compared to inhaled beclometasone alone in primary or exploratory outcome measures.

### Changes in inflammatory biomarkers

After 4 and 8 weeks, the total cell count and differential cell counts in induced sputum were similar for atorvastatin and placebo (Table 3). The sputum and serum concentrations of inflammatory cytokines and mediators were similar after atorvastatin compared to after placebo treatment at the 4 week and 8 week time points (Table 3 and data not shown). There was no significant change in FE_NO _after atorvastatin compared to placebo (data not shown).

**Table 3:** Inflammatory outcomes after atorvastatin treatment or placebo

Variables	Baseline		4 weeks		Treatment difference, atorvastatin minus placebo (95% CI)	8 weeks		Treatment difference, atorvastatin minus placebo (95% CI)
				
	Atorvastatin (n = 35)	Placebo (n = 32)	Atorvastatin (n = 31)	Placebo (n = 29)		Atorvastatin & ICS (n = 32)	Placebo & ICS (n = 24)	
**Sputum differential** **cell count &** **proportion (%)**								

**Total cell count (×10^4^)**	651.5 (547.0, 890.5)	620.8 (543.2, 758.8)	628.5 (533.5, 921.5)	602.0 (512.0, 791.0)	43.0 (-75.2, 161.1) p = 0.469	607.8 (517.0, 785.5)	658.5 (563.2, 860.2)	-81.6 (-177.2,14.0) p = 0.093

**Macrophage (×10^4^)** **Macrophage %**	154.5 (111.5, 263.0) 38.4 (29.6, 61.3)	179.8 (87.2, 242.2) 45.1 (31.8, 58.7)	159.5 (132.0, 221.0) 45.5 (31.5, 58.9)	142.5 (81.0, 220.0) 38.5 (24.6, 54.6)	16.9 (-27.5, 61.3) p = 0.450 4.8 (-5.9, 15.5) p = 0.374	173.2 (126.2, 247.2) 41.4 (34.1, 54.9)	174.2 (122.8, 250.5) 44.2 (32.3, 59.0)	-3.0 (-47.6, 41.6) p = 0.893 -0.5 (-9.6, 8.6) p = 0.913

**Neutrophil (×10^4^)** **Neutrophil %**	131.0 (76.0, 232.5) 37.8 (18.3, 54.2)	122.8 (56.2, 201.2 29.9 (15.0, 49.1)	136.5 (61.0, 233.5) 33.6 (18.2, 55.8)	99.0 (69.5, 299.5) 31.5 (17.6, 63.2)	-19.2 (-72.0, 33.6) p = 0.470 -4.7 (-15.7, 6.4) p = 0.401	151.8 (80.5, 211.5) 38.3 (23.0, 52.8)	149.0 (78.2, 229.0) 34.9 (20.7, 49.5)	-8.0 (-49.4, 33.4) p = 0.700 2.1 (-8.0, 12.1) p = 0.680

**Eosinophil (×10^4^)** **Eosinophil %**	3.0 (0.5, 7.5) 0.6 (0.1, 2.4)	1.5 (0.0, 5.8) 0.4 (0.0, 1.4)	1.5 (0.0, 4.5) 0.4 (0.0, 1.0)	1.5 (0.0, 3.5) 0.3 (0.0, 1.2)	-3.2 (-8.6, 2.3) p = 0.253 -1.0 (-2.3, 0.3) p = 0.138	1.3 (0.3, 5.5) 0.4 (0.1, 1.1)	0.8 (0, 2.5) 0.2 (0, 0.6)	0.4 (-1.6,2.5) p = 0.677 0.1 (-0.4, 0.5) p = 0.798

**Lymphocyte (×10^4^)** **Lymphocyte %**	1.5 (0.0, 3.0) 0.3 (0.0, 0.7)	0.8 (0.0, 2.8) 0.2 (0.0, 0.7)	0.5 (0.0, 3) 0.1 (0.0, 0.7)	0.5 (0.0, 1.5) 0.1 (0.0, 0.5)	0.8 (-0.2, 1.9) p = 0.125 0.1 (-0.2, 0.4) p = 0.425	0.8 (0.0, 2.0) 0.2 (0.0, 0.7)	1.3 (0.0, 2.5) 0.4 (0.0, 0.6)	-0.1 (-1.3, 1.1) p = 0.865 0.1 (-0.3, 0.4) p = 0.757

**Bronchial epithelial cell (×10^4^)** **Br. epithelial cell %**	44.0 (26.0, 90.5) 11.5 (5.9, 26.0)	49.5 (29.0, 117.0) 12.3 (7.8, 29.2)	33.0 (18.0, 78.0) 8.4 (5.9, 17.8)	37.5 (24.0, 89.5) 9.2 (5.6, 24.8)	20.6 (-9.6, 50.9) p = 0.177 1.6 (-5.9, 9.0) p = 0.680	40.0 (28.0, 75.8) 11.2 (6.8, 22.2)	57.8 (39.5, 92.8) 13.3 (9.2, 22.5)	-10.3 (-32.7, 12.1) p = 0.361 -1.3 (-7.7, 5.1) p = 0.679

**Sputum mediators**								

**Leukotriene B_4_** **(pg/ml)**	103.1 (79.7, 185.4)	172.0 (106.0, 422.4)	98.4 (56.9, 215.2)	175.2 (77.0, 336.0)	-49.4 (-205.1, 106.4) p = 0.528	104.9 (41.9, 151.5)	159.0 (81.0, 520.2)	-231.4 (-589.5, 126.6) p = 0.200

**MPO** **(ng/mL)**	16.5 (6.4, 39.6)	27.0 (10.6, 40.6)	16.4 (9.0, 30.5)	20.6 (10.6, 34.1)	-16.5 (-48.0, 15.0) p = 0.298	14.8 (6.4, 34.5)	28.1 (8.9, 68.8)	-111.2 (-264.8, 42.5) p = 0.152

**Interleukin-6 (pg/ml)**	139.5 (33.9, 276.5)	220.4 (70.0, 498.5)	109.3 (33.9, 351.5)	144.4 (40.3, 391.6)	-5.6 (-254.1, 243.0) p = 0.964	147.2 (35.9, 223.0)	122.0 (34.1, 518.6)	-22.5 (-247.3, 202.3) p = 0.841

**Interleukin-8 (pg/ml)**	813.1 (238.8, 3000.0)	3000.0 (426.1, 3000.0)	408.5 (179.7, 3000.0)	1686.0 (353.2, 3000.0)	-453.4 (-1071.9, 165.1) p = 0.127	386.5 (180.7, 3000.0)	541.5 (239.4, 3000.0)	172.3 (-600.8,945.4) p = 0.656

**Interleukin-10 (pg/ml)**	13.6 (1.0, 126.9)	13.0 (1.2, 80.9)	1.8 (0.3, 105.3)	15.4 (0.8, 53.4)	27.5 (-53.2, 108.2) p = 0.497	6.1 (0.7, 72.6)	4.6 (0.7, 62.9)	-4.2 (-132.8, 124.5) p = 0.948

**Serum biomarkers**								

**hs-CRP (mg/L)**	2.8 (1.6, 4.0)	3.6 (1.5, 6.9)	2.6 (1.6, 5.0)	2.8 (1.4, 5.6)	0.9 (-4.1, 5.9) p = 0.718	1.9 (1.0, 4.1)	2.1 (1.0, 5.1)	0.6 (-2.8, 3.9) p = 0.731

**Interleukin-6 (pg/ml)**	2.0 (0.2, 4.1)	2.6 (0.2, 30.0)	2.7 (0.2, 11.0)	1.8 (0.2, 26.0)	-9.1 (-20.8, 2.7) p = 0.127	0.4 (0.2, 8.8)	3.1 (0.2, 21.2)	16.7 (-29.5, 62.8) p = 0.472

Abbreviations: CRP, C-reactive protein; MPO, myeloperoxisade; Mediator levels pg/ml unless otherwise indicated. Data represented as median (IQR) unless otherwise indicated.

### Change in biochemical markers

The biochemical effects of atorvastatin therapy were reflected in significant reduction in concentration of serum lipids; cholesterol [-1.55, 95% CI -1.88 to -1.23 P < 0.0001], LDL-cholesterol [-1.50, 95% CI -1.88 to -1.13 P < 0.0001] and triglyceride [-0.49, 95% CI -0.87, -0.11 P = 0.013] (Table 4).

**Table 4:** Serum biochemical outcomes after atorvastatin treatment or placebo

Variables	Baseline		8 weeks		Treatment difference, atorvastatin minus placebo (95% CI)
		
	Atorvastatin (n = 35)	Placebo (n = 32)	Atorvastatin & ICS (n = 32)	Placebo &ICS (n = 25)	
**Total cholesterol** **(mmol/l)**	5.15 (0.89)	5.67 (1.12)	3.62 (0.76)	5.34 (0.90)	-1.55 (-1.88, -1.23) **P < 0.0001**

**Triglycerides** **(mmol/l)**	1.59 (0.87)	2.18 (1.41)	1.02 (0.35)	1.74 (1.29)	-0.49 (-0.87, -0.11) **P = 0.013**

**LDL-cholesterol** **(mmol/l)**	3.19 (0.82)	3.49 (0.99)	1.89 (0.62)	3.46 (0.87)	-1.50 (-1.88, -1.13) **P < 0.0001**

**HDL-cholesterol** **(mmol/l)**	1.24 (0.35)	1.26 (0.31)	1.29 (0.35)	1.23 (0.27)	0.03 (-0.06, 0.13) p = 0.497

**Alkaline phosphatase** **(IU/l)**	83.33 (31.36)	75.16 (21.04)	98.62 (91.26)	77.16 (19.58)	5.94 (-15.67, 27.54) p = 0.584

**ALT** **(IU/l)**	17.58 (9.27)	22.50 (11.88)	22.00 (14.37)	24.04 (11.70)	3.67 (-1.84, 9.18) p = 0.188

Abbreviations: ALT, alanine aminotransferase

Data represented as mean (SD).

### Adverse events

No suspected unexpected serious adverse reactions (SUSARs) were recorded. Adverse events (AEs) were similar between treatment groups. Two serious adverse events occurred, one in each group and neither related to study drug. For adverse events greater than 5% in frequency, the only severe AE was back pain in 3 (8.3%) subjects in the atorvastatin group versus 0 in the placebo group. Moderate side effects were more frequent in the atorvastatin group: oral candidiasis (2 subjects versus 0), neck pain (2 versus 0) and asthma worsening (2 versus 1).

### Compliance

Compliance assessed by inhaler weight and tablet count was greater than 90% in both groups. The marked reduction in LDL-cholesterol in the group receiving atorvastatin (Table 4) provides further evidence of compliance. The weights of the returned inhalers indicate similar compliance with the inhaled beclometasone treatment.

## Discussion

In this randomized controlled study we found no improvement in the atorvastatin group compared to the placebo group in lung function in smokers with mild to moderate asthma, whereas there was an improvement in ACQ and AQLQ scores at 4 weeks. Atorvastatin treatment had no anti-inflammatory effects on induced sputum cell counts or on selected sputum or circulating blood mediator levels.

Three previous randomized controlled crossover clinical trials reported an absence of clinical benefit with short-term statin therapy on asthma symptoms or lung function in non-smokers with asthma[9-11]. The lack of improvement in PEF, FEV_1 _or airway responsiveness to methacholine in asthmatic smokers following atorvastatin supports the conclusions of these earlier studies that statins do not have a short-term beneficial effect on lung function in asthma irrespective of smoking status.

In addition to poor symptom control, smokers with asthma have worse asthma-specific quality of life scores[31]. The AQLQ is a well validated score of subjective well being and improving this index is an important goal of asthma management[32]. As one of a number of outcome measure we found a mean between-group difference in change from baseline in AQLQ score of 0.52 at 4 weeks, which is just greater than the minimal clinically significant change in score of 0.5[33]. The individual AQLQ domains for symptoms and emotions also crossed this threshold. The improvement in AQLQ score with atorvastatin is comparable to that reported with long-acting β_2 _agonist therapy[34] and with leukotriene receptor antagonist therapy in non-smokers with asthma[35]. The non-significant mean improvement in ACQ score of -0.37 did not reach the minimum important reduction threshold of -0.5[36]. Improvements in subject-centered outcomes with interventions in asthma are usually associated with better lung function, although this is not always the case. For example, clinically important improvements in the AQLQ score were observed in a trial comparing different formulations of inhaled beclometasone[37] and in a double-blind sham-controlled trial of bronchial thermoplasty[38] while in both studies conventional clinical indices of lung function and asthma control were similar.

Statins restore corticosteroid sensitivity in patients with asthma[14,15], possibly by restoring HDAC activity[16] or by increased induction of indoleamine 2, 3-dioxygense resulting in increased secretion of the anti-inflammatory cytokine IL-10[15]. Statins also inhibit IL-17 expression and secretion from human Th17 cells[39], which have been implicated in causing corticosteroid resistance[40]. Based on these studies we wished to determine whether atorvastatin might restore sensitivity to corticosteroids and had hoped to observe a beneficial effect of atorvastatin and inhaled beclometasone compared to inhaled beclometasone alone, but found no significant effect in outcome measures at the 8 week time point. It is possible that 4 weeks was not long enough to demonstrate a synergistic effect, although in previous studies a 4 week treatment period was long enough to show the potentiating effects of low dose theophylline on corticosteroid sensitivity in smokers with asthma[41].

Statins have pleiotropic properties including anti-inflammatory and immuno-modulatory effects [5] that may be beneficial in the treatment of chronic inflammatory airway diseases[6]. The anti-inflammatory effects of statins are thought to be mediated by inhibition of the isoprenylation of small G-protein signalling molecules and by prevention of lipid raft formation, pathways involved in cell proliferation, activation and oxidative stress. Statins have immune modulating properties in animal models of allergic asthma[7,8] and COPD[12,13]. In our study, a limited investigation of circulating and airway inflammatory cells and mediators failed to show any effects of atorvastatin therapy on inflammatory biomarkers including CRP, despite good drug compliance and significant reduction of serum lipid markers.

There are several limitations of the study. Firstly, if fewer than three days of data were recorded during the seven days immediately preceding each study visit, patient diary entries for morning PEF were substituted in place of the missing value, which raises the possibility of increased variability in PEF measurements. However this procedure was performed in a minority of PEF measurements used in the analysis and is unlikely to have influenced the PEF result. Secondly, while questionnaire scores were not significantly different at baseline, it is possible that the slight difference in the proportion of participants taking LABA prior to screening may have had an effect on subsequent measurements of asthma control. The short duration of treatment with atorvastatin may have missed an effect on clinical or inflammatory outcomes. Future studies should assess whether treatment with statins influence outcome measures such as exacerbation rate and decline in lung function in smokers with asthma and in individuals with more severe disease. Statins have an inhibitory effect on human airway smooth muscle cell proliferation[42] and indices of airway remodelling[43-45]. It is possible that the administration of atorvastatin therapy for a longer duration of time may improve outcome measures of airway remodelling in asthma'

## Conclusion

In conclusion, short-term treatment with atorvastatin does not improve lung function in smokers with mild to moderate asthma, but may improve asthma quality of life. Further trials using statins in asthmatic smokers are indicated.

## Competing interests

The authors declare that they have no competing interests.

## Authors' contributions

GB: Contributed to the acquisition and interpretation of the data; RC: Contributed to the acquisition and interpretation of the data and drafted the manuscript for important intellectual content; CMcS: Contributed interpretation of the data, carried out the immunoassays and drafted the manuscript for important intellectual content; MB: Contributed to the acquisition of the data; CW: Participated in the design of the study and performed the statistical analysis; ID: Carried out the immunoassays; LJ: Carried out the immunoassays; JL: Contributed to the acquisition of the data; SL: Performed the statistical analysis; MS: Contributed to interpretation of the data and drafted the manuscript for important intellectual content; FM: Contributed to the acquisition and interpretation of the data; NCT: Conceived the study, participated in its design, contributed to interpretation of the data and drafted the manuscript for important intellectual content. All authors read and approved the final manuscript.

## Pre-publication history

The pre-publication history for this paper can be accessed here:

http://www.biomedcentral.com/1471-2466/11/16/prepub
